# Simulation of solute transport behaviors in saturated karst aquifer system

**DOI:** 10.1038/s41598-021-94950-7

**Published:** 2021-08-02

**Authors:** Xuewei Chu, Hanghang Ding, Xuemei Zhang

**Affiliations:** 1 College of Resource and Environmental Engineering, Guizhou University, Guiyang, 550025 China; 2 College of Geoscience and Surveying Engineering, China University of Mining & Technology (Beijing), Beijing, 100083 China; 3 National Engineering Research Center of Coal Mine Water Hazard Controlling, Beijing, 100083 China; 4 Non-Ferrous Metals and Nuclear Industry Geological Exploration Bureau Geophysical and Geochemical Exploration Team of Guizhou, Duyun, 558000 China

**Keywords:** Environmental sciences, Hydrology

## Abstract

The karst development makes aquifer have strong anisotropy and heterogeneity. In order to reveal the characteristics of solute transport in the karst fissure–conduit aquifer system, this study presents a physical model of fissure–conduit in laboratory experiments to carry out the solute transport simulation. In this paper, the tracer tests of fissure–conduit combination, fissure, and conduit solute transport process in saturated flow are designed. We found that different aquifer structures and tracer injection points have an influence on the shape of the breakthrough curve. Besides, the two-dimensional dispersion model of tracer injection of the instantaneous point was used to calculate the dispersion parameters of each group of experiments. Then, the dynamic responses of the linear distance (*x*) between the injection point and the receiving point, initial time (*t*_0_), peak time (*t*_m_), peak concentration (*c*_m_), average tracer transport velocity (*V*), and porosity (*p*) of aqueous media to the longitudinal dispersion coefficient are discussed. In addition, according to the measured data, Gaussian multi-peak fitting can be used to reflect the overall shape and change trend of the multi-peak BTC. These results demonstrate the solute transport behaviors in the saturated karst aquifer system, which have important reference significance for solving the engineering environmental problems in the karst area.

## Introduction

In recent decades, human activities have caused a large area of groundwater pollution. Understanding the movement of pollutants in groundwater is a prerequisite for controlling groundwater pollution^1,2^. However, the groundwater seepage characteristics of different aquifer media are extremely different. Due to the development of underground karst fissures and conduits, the transport characteristics of karst groundwater are also different from that of groundwater in homogeneous pore media, resulting in complicated seepage characteristics and extremely complex changes in solute transport rules^3,4^. Therefore, the prediction of solute transport rate, diffusion mechanism, and distribution range becomes more difficult because of the influence of complex aquifer structures^5,6^. Studying the process of solute transport in karst aquifer media is the key to solving engineering environmental problems in karst areas.

In recent years, the one-dimensional pipe flow model with variable gap width pointed out that the flow of groundwater in fissures flows along many curved grooves, which leads to the multi-peak phenomenon of solute penetration curve^7,8^. Then, Field and Leij^9^ successfully applied the dual-advection dispersion equation to the tracer test of the karst aquifer composed of two connected but mostly separated pipes, which proved the suitability of using multiple dispersion models when conditions permit. Morales et al.^6^ studied the influence of the geometry of the conduit detention area on the shape and evolution of the breakthrough curve (BTC) under different hydrological conditions. A similar physical simulation experiment has become an effective means to study the solute transport mechanism of karst groundwater. In China, much more attentions also have been focused on it to study solute transport. Some simulation experiments showed that there is an exponential relationship between seepage flow and effective porosity, fissure density, fissure occurrence, number of inlet and outlet, and there is an exponential relationship between solute flux and fissure density^10^. The influence of the doline on the flow in the fissure network is inversely proportional to the distance between the fissure and the doline. Only when the concentrated supply intensity of the doline is far greater than the scattered supply intensity of the fissure, the doline will supply the fissure^11^. In addition, the physical model test can also be used to analyze the influence of geometric parameters (pipe diameter and connection mode) on the dispersion. Liu et al.^12^ found that the pipe diameter has little influence on the equivalent dispersion, while the length of pipe path, number difference, and pipe diameter difference has a great influence on the equivalent dispersion, and the number of pipes and pipe surface has a positive correlation with the equivalent dispersion.

The above research and discovery of the simulation experiment of the fissure–conduit have important reference significance for understanding the solute transport in the karst aquifer medium. However, there are relatively few indoor simulation studies that consider rainfall recharge, epikarst zones, dolines, fissures, conduits, and springs based on the field with highly similar aquifer structure^13–15^. Therefore, a similar physical simulation experiment is necessary to further study the solute transport in the complex karst aquifer medium.

**Figure 1:**
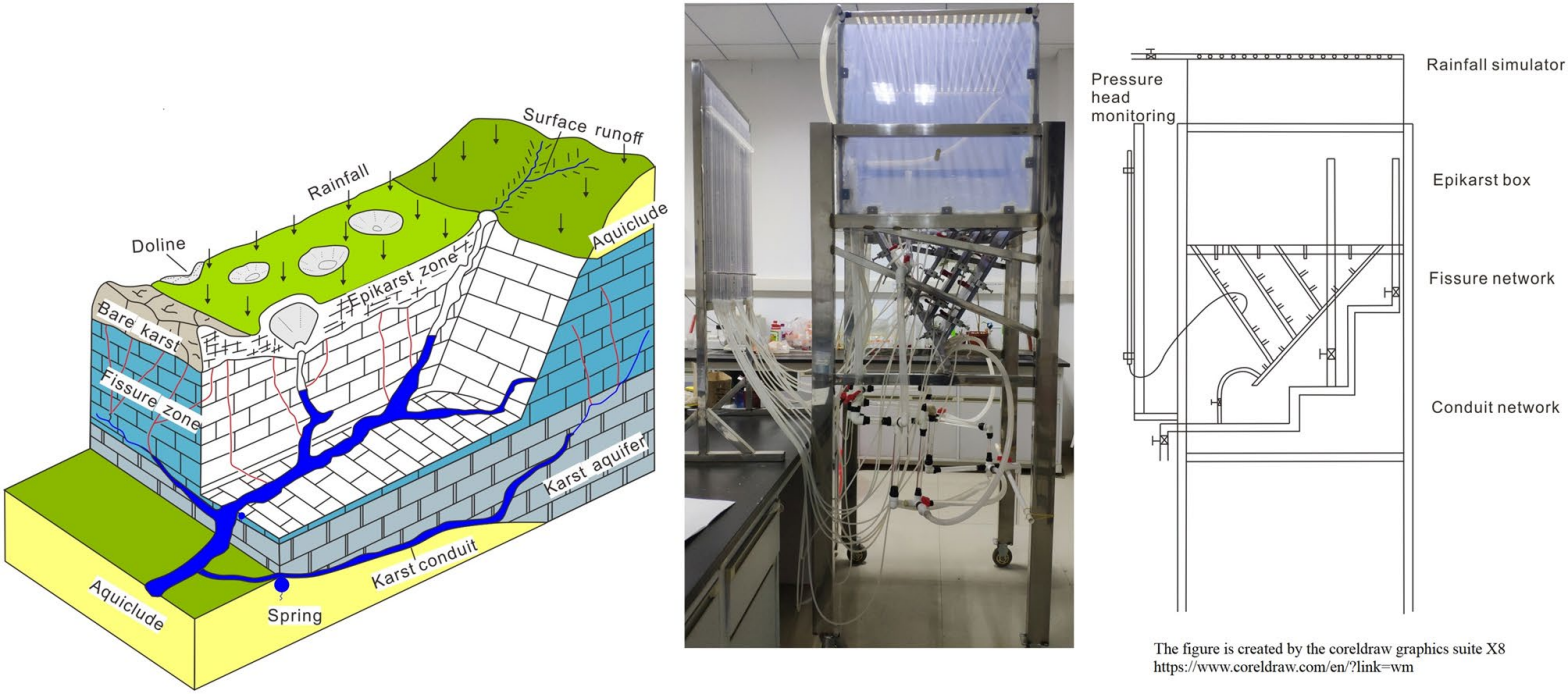
Conceptual model and physical model of karst fissure–conduit system^14^.

In response of these problems, we have verified a physical model considering the hydrological cycle process of the karst aquifer system by analyzing the conceptual model and establishing a similar physical model^14^. The established model includes hydrological elements such as rainfall recharge, doline, epikarst, fissures, conduits, and springs (Fig. 1). Moreover, the hydrologic process under different rainfall conditions and different aquifer structures was designed. Importantly, the experiment proved the rationality of the model structure design.

In this study, we used the same physical model to simulate the solute transport process and explore solute transport process in the aquifer medium of fissure–conduit. Meantime, the rainfall intensity is controlled by the rainfall simulator. When the water flow is stable, a certain concentration of tracer is injected at the selected tracer point to simulate the solute transport at different positions. Subsequently, samples were taken regularly at the outlet of the pipeline to determine the concentration and plot the BTC of solute transport.

The transport and prediction of pollutants in the groundwater system are based on the hydrodynamic dispersion equations. In karst areas, through the study of hydrodynamic dispersion, the karst leakage paths, seepage types, pollutant transport, and change characteristics of a certain area can be found^16,17^. Dispersion parameters are an important basis for the study of solute transport process and the water quality prediction. Therefore, the method of determining dispersion parameters has become a crucial point. At present, the most reliable method for determining dispersion parameters is tracer test, which mainly includes field tracer test and indoor tracer test, while the indoor tracer test is an effective method for quantitative control^18,19^. A quantitative tracing test is a powerful tool, which can not only determine the hydraulic connection between the two points but also provide direct information on the trajectory of groundwater movement, as well as the BTC, from which the solute transport in karst aquifer can be obtained^20,21^.

To begin with, we found that three kinds of aquifer structure and saturation conditions, fissure–conduit, fissure, and conduit, have an influence on the BTC of solute transport, mainly in the shape of the curve, the initial time of tracer transport, peak time, and duration. Then, the two-dimensional dispersion model of the instantaneous point injection of the tracer was used to calculate the dispersion parameters of each group of experiments, so as to analyze the factors affecting the longitudinal dispersion coefficient of solute transport, including the linear distance (*x*), the initial time (*t*_*0*_), the peak time (*t*_*m*_), the peak concentration (*c*_*m*_), the tracer transport velocity (*V*) and the porosity of aquifer medium (*p*). In addition, in different aquifer structures, each influencing factor has a different degree of influence on the longitudinal dispersion coefficient. Finally, when multi-point injection is performed in the saturated flow, the breakthrough curve appears multi-peak phenomenon. Furthermore, the Gaussian multi-peak fitting method can be used to predict the overall shape and change trend of the curve. To sum up, it is quite necessary to grasp the transport process of karst water, whether it is to use or protect karst groundwater. In this paper, the simulation and analysis of solute transport in aquifer media of fissure–conduit has very important reference significance for the study of groundwater dynamics and the solution of pollutant transport in karst areas.

## Materials and methods

### Theoretical background

Hydrodynamic dispersion includes molecular diffusion affected by concentration gradient and mechanical dispersion caused by uneven flow velocity. When fluid flows in porous media, the interaction between solid and liquid phases is very complex, including the adsorption, precipitation, dissolution, ion exchange, chemical reaction, and biological process of tracer particles on solid surface^1,22^. However, mechanical action is the most important factor for tracer transport. Because of the existence of pore system, the velocity distribution in the pore is not uniform regardless of its size and direction. The mechanical dispersion of water flow is mainly considered in this tracer test.

Under saturated aquifers, the mathematical model of the two-dimensional dispersion of instantaneous point-injection tracer can be expressed as follows:

1 $$\left\{ {\begin{array}{*{20}l} {\frac{{\partial c}}{{\partial t}} = D_{L} \frac{{\partial ^{2} c}}{{\partial x^{2} }} + D_{T} \frac{{\partial ^{2} c}}{{\partial y^{2} }} - V\frac{{\partial c}}{{\partial x}}} \hfill & { - \infty < x;y < \infty ;t > 0} \hfill \\ {c\left( {x,y,0} \right) = 0} \hfill & { - \infty < x;y < \infty } \hfill \\ {\mathop \smallint \limits_{{ - \infty }}^{\infty } \mathop \smallint \limits_{{ - \infty }}^{\infty } ncdxdy = m} \hfill & {t > 0} \hfill \\ {c\left( {x,y,t} \right)|_{{(x^{2} + y^{2} ) \to \infty }} = 0} \hfill & {t > 0} \hfill \\ \end{array} } \right.$$

where *c* is the tracer concentration; D_L_ is the longitudinal dispersion coefficient; *D*_T_ is the transverse dispersion coefficient; *V* is the flow velocity; *m* is the injection mass of the tracer; *n* is the porosity; *x*, *y* is the coordinate of any point in the flow field.

The solution to this model^23^ is:

2 $$c\left( {x,y,t} \right) = \frac{{m/n}}{{4\pi t\sqrt {D_{{\text{L}}} D_{{\text{T}}} } }}exp\left[ { - \frac{{\left( {x - Vt} \right)^{2} }}{{4D_{{\text{L}}} t}} - \frac{{y^{2} }}{{4D_{{\text{T}}} t}}} \right]$$

Following is a brief introduction to the principle and procedure of straight-line graphic method for solving dispersion parameters^24^.

First derivation of *t* in Eq. (2) can be written as follows:

3 $${c}^{{\prime}}\left(x,y,t\right)=-\frac{m}{{4\pi nt}^{2}\sqrt{{D}_{\mathrm{L}}{D}_{\mathrm{T}}}}\left(\frac{{x}^{2}-{V}^{2}{t}^{2}}{{4D}_{\mathrm{L}}t}+\frac{{y}^{2}}{4{D}_{\mathrm{T}}t}-1\right)exp\left[-\frac{{\left(x-Vt\right)}^{2}}{4{D}_{\mathrm{L}}t}-\frac{{y}^{2}}{4{D}_{\mathrm{T}}t}\right]$$

For a point (x,y) of the solute transport space, if the point concentration peaks at the t time, then the first derivative ${c}^{{\prime}}\left(x,y,t\right)$=0 of Eq. (3)

With $\frac{m}{{4\pi nt}^{2}\sqrt{{D}_{\mathrm{L}}{D}_{\mathrm{T}}}}\ne 0$, $exp\left[-\frac{{\left(x-Vt\right)}^{2}}{4{D}_{\mathrm{L}}t}-\frac{{y}^{2}}{4{D}_{\mathrm{T}}t}\right]\ne 0$. we have

4 $$\frac{{x}^{2}-{V}^{2}{t}^{2}}{4{D}_{\mathrm{L}}t}+\frac{{y}^{2}}{4{D}_{\mathrm{T}}t}-1=0$$

Then, the time of the maximum concentration (*t*_m_) is:

5 $${t}_{\mathrm{m}}=\frac{{D}_{\mathrm{L}}}{{V}^{2}}\left[\sqrt{4+{V}^{2}\left(\frac{{x}^{2}}{{D}_{\mathrm{L}}^{2}}+\frac{{y}^{2}}{{D}_{\mathrm{L}}{D}_{\mathrm{T}}}\right)}-2\right]$$

According to Eqs. (2) and (5), the maximum concentration value can be obtained as:

6 $${c}_{\mathrm{m}}\left(x,y,t\right)=-\frac{m}{4\pi n{t}_{\mathrm{m}}\sqrt{{D}_{\mathrm{L}}{D}_{\mathrm{T}}}}exp\left[-\frac{{(x-V{t}_{\mathrm{m}})}^{2}}{4{D}_{\mathrm{L}}{t}_{\mathrm{m}}}-\frac{{y}^{2}}{4{D}_{\mathrm{T}}{t}_{\mathrm{m}}}\right]$$

For $y=0$, Eq. (4) can be simplified as:

7 $${x}^{2}-{V}^{2}{t}_{\mathrm{m}}^{2}-4{t}_{\mathrm{m}}{D}_{\mathrm{L}}=0$$

We can get the following equation based on Eq. (7)

8 $${D}_{\mathrm{L}}=\frac{{x}^{2}-{V}^{2}{t}_{\mathrm{m}}^{2}}{4{t}_{\mathrm{m}}}$$

Therefore, as *V* is known, the *D*_L_ can be calculated using Eq. (8). As *V* is un known, Eq. (8) can also be written as follows to calculate the *D*_L_ by parameter method.

9 $${D}_{\mathrm{L}}=\frac{{x}^{2}}{4{t}_{\mathrm{m}}\left(\frac{{V}^{2}{t}_{\mathrm{m}}^{2}}{{x}^{2}-{V}^{2}{t}_{\mathrm{m}}^{2}}+1\right)}$$

In order to simplify the above expression, the function *k* is defined as:

10 $$k=\frac{{V}^{2}{t}_{\mathrm{m}}}{{x}^{2}-{V}^{2}{t}_{\mathrm{m}}^{2}}$$

Then, Eq. (9) can be written as:

11 $${D}_{\mathrm{L}}=\frac{{x}^{2}}{4{t}_{\mathrm{m}} (k{t}_{\mathrm{m}}+1)}$$

By using Eq. (10) which was obtained

12 $$\mathrm{V}=\sqrt{\frac{k({x}^{2}-{V}^{2}{t}_{\mathrm{m}}^{2})}{{t}_{\mathrm{m}}}}$$

By applying Eq. (8) to (12), *V* can be written as:

13 $$\mathrm{V}=2\sqrt{k{D}_{\mathrm{L}}}$$

When the *K* is known, we can obtain the *D*_L_ and *V*. Here, the following introduces the straight-line slope method to find *K.*

For $y=0$, by integrating Eqs. (6) and (2), we can obtain

14 $$ln\frac{{\mathrm{c}}_{\mathrm{m}}{t}_{\mathrm{m}}}{ct}=-\frac{{\left(x-V{t}_{\mathrm{m}}\right)}^{2}}{4{D}_{\mathrm{L}}{t}_{\mathrm{m}}}+\frac{{(x-Vt)}^{2}}{4{D}_{\mathrm{L}}t}$$

By applying Eq. (8) to (14), we can obtain

15 $$ln\frac{{\mathrm{c}}_{\mathrm{m}}{t}_{\mathrm{m}}}{ct}+\frac{t-{t}_{\mathrm{m}}}{t}=\frac{{V}^{2}{t}_{\mathrm{m}}}{{x}^{2}-{V}^{2}{t}_{\mathrm{m}}^{2}}\times \frac{{(t-{t}_{\mathrm{m}})}^{2}}{t}$$

Here, assuming the $\mathrm{X}=\frac{{(t-{t}_{\mathrm{m}})}^{2}}{t}$, and $\mathrm{Y}=ln\frac{{\mathrm{c}}_{\mathrm{m}}{t}_{\mathrm{m}}}{ct}+\frac{t-{t}_{\mathrm{m}}}{t}$, the Eq. (14) as

16 $$\mathrm{Y}=kX$$

Thus, Eq. (16) is a straight line in rectangular coordinate system, and *K* is the slope of the straight line.

### Experiment model

The model consists of the rainfall simulator, the infiltration box of epikarst, the fissures zone and, the conduits zone. In this study, first, the rainfall simulator is used to simulate the natural rainfall in the karst area. Second, the epikarst is brought into the infiltration box to simulate the centralized infiltration supply of the doline and the decentralized infiltration supply of the surface fissures zone in the karst area. Third, the fissure zone is mainly used to simulate the flow of water passing through a group of parallel water-conducting fissure zones and then being cut by a major fissure and converging into the major fissure, and finally discharged at the bottom of the major fissure; Forth, the conduit zone is mainly used to simulate the centralized drainage conduit system after the multi-source confluence, which is vertically divided into three steps. The one step mainly receives the water supply from the doline and the surface layer after the infiltration confluence; another step mainly receives the drainage supply from the fissure zone; the final step mainly simulates the spring point's exposure on the erosion datum plane. Also, the conduit zone in the vertical direction is like a branch with many branches (Fig. 1). The detailed model design parameters and model structure can obtain from past research^14^.

### Experiment process

#### Tracer

**Figure 2:**
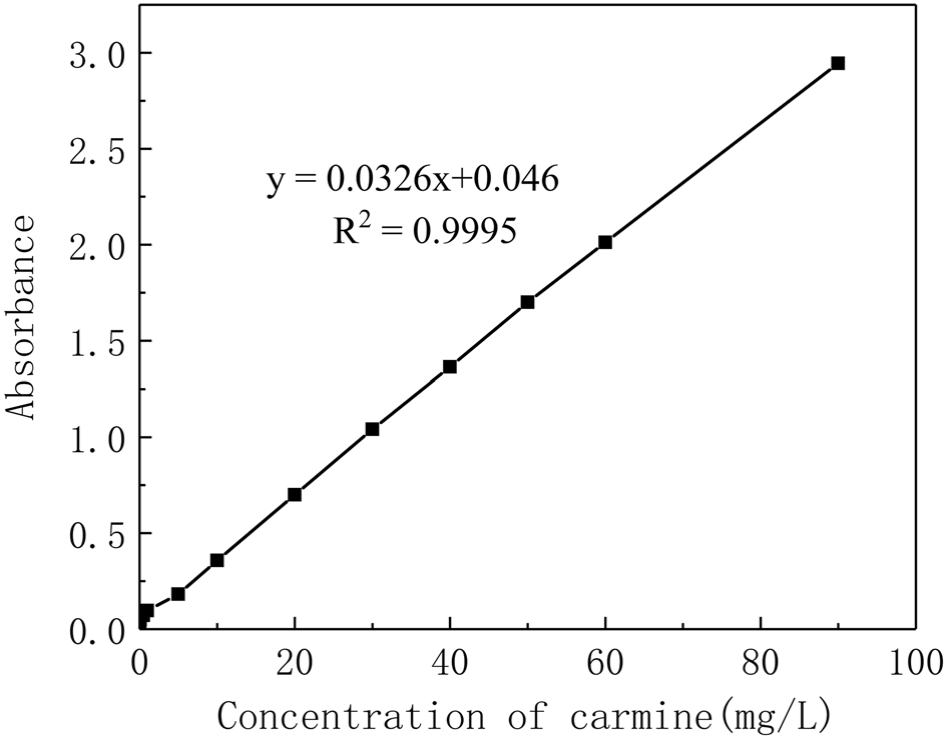
The standard curve of dye solution concentration-absorbance.

Dye tracing is an effective method to describe the flow characteristics of groundwater in Karst aquifers^25,26^. In this experiment, we chose the carmine as a tracer. Therefore, the concentration of carmine reserved liquid is configured (the concentration is 3 g/L). Using the reserved liquid by diluting different times, to measure its absorbance value with a spectrophotometer and draw the standard curve (Fig. 2).

#### Solute transport

Before the experiment, we should reduce the inner diameter of the discharge pipe at the outlet, and control the flow of water supply, so that the water flow can fill the lower fissure zone and conduit zone, and make it saturated. Under the condition of the full water of the fissure–conduit, the fissure, and the conduit, different injection points were selected for the tracing test. In the experiment, the injection amount of all tracers was controlled at 20 ml. However, some experiments were difficult to be evenly distributed. In order to achieve reliable experimental results, the injection amount of tracers was increased appropriately. In terms of current research problems, it does not affect the analysis of experimental results.

1. Install the submersible pump in the water supply tank and connect with the water inlet of the rainfall simulator.
2. Open the water valve at the water supply pipe of the rainfall simulator to supply water to the rainfall simulator after opening all water transport channels in the fissure–conduit.
3. When the epikarst zone is not filled, it can be observed that the water flows down at the joint of the fissure and the conduit, and then flows through the fissure–conduit zone, which discharges at the spring outlet after rainwater falls to the bottom of the surface karst box.
4. Monitor the flow at the outlet, and prepare the tracer for the next step when the flow is stable.
5. Take the prepared carmine solution from the syringe with a measuring range of 20 ml, inject it into the selected tracer injection point instantaneously, and record the sampling time.
6. Observe the movement of carmine in the model. When carmine is near the outlet, start to take samples. During the period when the color is deepened, take more samples. The time interval is controlled within 3–5 s. When the concentration changes slowly, the sampling interval can be extended to about 10–30 s. When the color is diluted and remains unchanged, the sampling interval can be extended to about 50 s.
7. When the color of the water flow at the outlet is almost the same as the background value, stop sampling.

## Results

We simulated three kinds of saturated flow solute transport experiments, which are the three kinds of water-filling scenarios. (1) fissure–conduit water-filling; (2) fissure water-filling; (3) conduit water-filling. The original inner diameter of the pipe outlet is 20 mm, and the flow state under the inner diameter is unsaturated flow. In the experiment, by changing the inner diameter of the pipe outlet, the lower part of the device is saturated with water, and adjusting the rainfall intensity at the same time, the spring flow at the outlet is stable. The inner diameter of the pipe outlet in the above three water saturation scenarios is 5 mm.

### Fissure–conduit water-filling (F–C–D)

**Figure 3:**
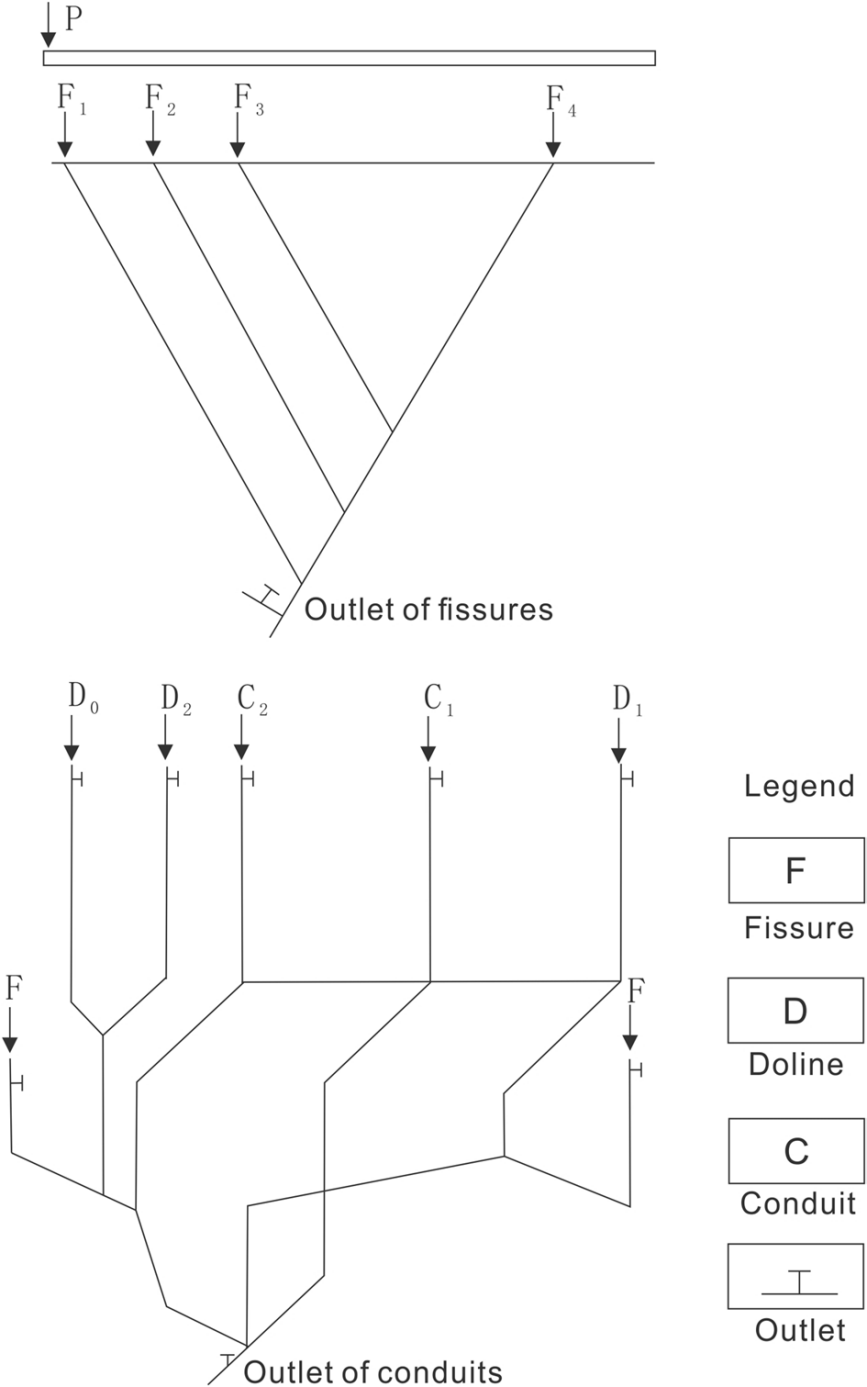
Injection points of fissures and conduits.

In this scenario, all the fissure–conduit water valves (F_1_, F_2_, F_3_, F_4_, C_1_, C_2_, D_0_, D_1_, D_2_) will be opened, and the stabilized rainfall intensity was 4.6744 mm/h and the outlet flow (*Q*) was 58.21 ml/s. For one thing, P, C_1_, C_2_, D_0_, D_1_, and D_2_ were selected for single-point injection (Fig. 3), and 20 ml tracer was put into each point.

**Figure 4:**
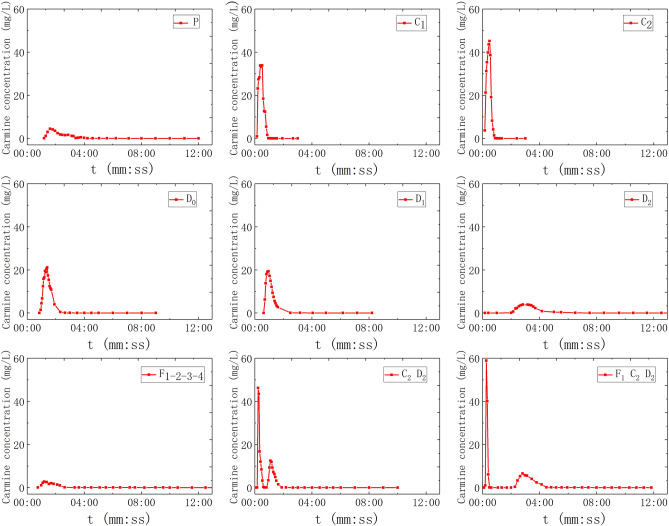
The BTC of solute transport under water-filling of fissure–conduit condition.

For another, F_1–2–3–4_, C_2_–D_2_ and F_1_–C_2_–D_2_ were carried out multiple points, wherein F_1–2–3–4_ means to install pipes with four fissures in parallel on one pipe, and to inject tracer (20 ml) at the inlet, C_2_–D_2_ means to inject tracer (10 ml) into C_2_ and D_2_ respectively at the same time. Moreover, F_1_–C_2_–D_2_ means to inject tracer (10 ml) into F_1_, C_2_, and D_2_ respectively at the same time. 9 groups of solute transport tests under the condition of saturated fissure–conduit were simulated, and the BTCs are shown in Fig. 4.

The BTC of single-point injection tracers are all single peak type, and there are both single-peak and multi-peak during multi-point injection (Fig. 4). The BTC of solute transport simulated by tracer injection at the entrance of rainfall simulator (P) and F_1–2–3–4_ are all single peak curves with a positive skew. In addition, BTCs have the characteristics of small peak concentration and long peak time. The tracer injection at point P will be uniformly dispersed by rainfall simulator and then fall to the top of the surface karst zone along with the rainwater. Meantime, it will be redistributed by the surface karst zone and flow into the aquifer medium of the fissure–conduit and discharged at the outlet. The process has experienced three times of redistribution, and the tracer injection has the longest transport path and long dilution time. Moreover, the BTC of fissure depicts that the water flow transport speed is slow under the condition of water-filling, which makes the tracer stay in the fissure longer. Therefore, the dilution effect on the concentration is strengthened, which makes the low peak concentration and the lag of peak time.

The shape of the BTC in the conduit and doline connected to the bottom of the epikarst box is similar to the symmetrical single peak type, in which the peak time of C_1_ and C_2_ is very short but the peak concentration is high. The order of catchment area of the three dolines is SD_0_ > SD_1_ > SD_2_, which correlates with the peak concentration and the relationship is *c*_m_D_0_ > *c*_m_D_1_ > *c*_m_D_2_. In the case of multi-point injection, (1) the peak concentration of F_1–2–3–4_ is all superposed which is single peak type; (2) C_2_–D_2_ is double peak type; and (3) F_1_–C_2_–D_2_ has a peak superposition showing double peak type, its second peak appearing time is later than the C_2_–D_2_, and the curve shape is wider and slower.

### Fissure water-filling (F)

**Figure 5:**
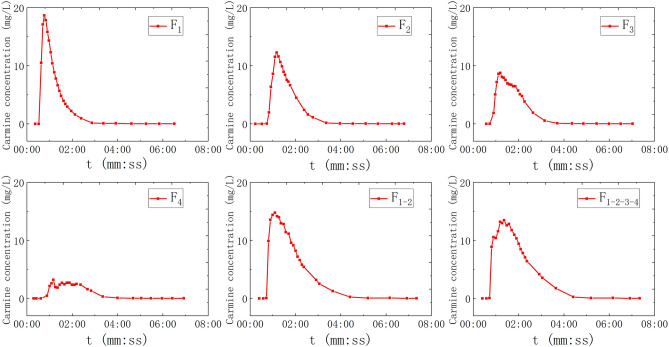
The BTC of solute transport under water-filling of fissure condition.

In this scenario, the water valve controlling the fissures (F1, F2, F3, F4,) will be opened and closed to control the water valves of all pipelines (C1, C2, D0, D1, D2), so that the water flow status is fissure saturated flow. The adjusted rainfall intensity was 3.9326 mm/h, and the outlet flow was 54.83 ml/s. As shown in Fig. 3, the point of F_1_, F_2_, F_3_, and F_4_ were selected for single-point injection (20 ml). F_1–2_ and F_1–2–3–4_ were selected for multi-point injection, wherein, 20 ml carmine solution put into the F_1–2_ and F_1–2–3–4_ respectively. A total of 6 sets of solute transport tests under the condition of fissure saturation were simulated in the experiments, and the BTCs are shown in Fig. 5.

In all tests of fissure saturated water flow, regardless of single-point injection or multi-point injection, the BTCs of solute transport is a single peak curve with positive skew, indicating that solute transport in each fissure is relatively synchronous. Figure 5 shows that the peak concentration decreased and the peak time-lagged in turn when F_1_, F_2_, F_3_, F_4_ was injected tracer at a single point respectively. The peak concentrations of F_1–2_ and F_1–2–3_ injected tracer at the same time, which is a single peak. The peak concentration of F_1–2_ is greater than F_1–2–3_, between F_1_ and F_2_. Compared with the BTCs of single-point injection, the curve shape of multi-point injection is wider and slower.

### conduit water-filling (C)

**Figure 6:**
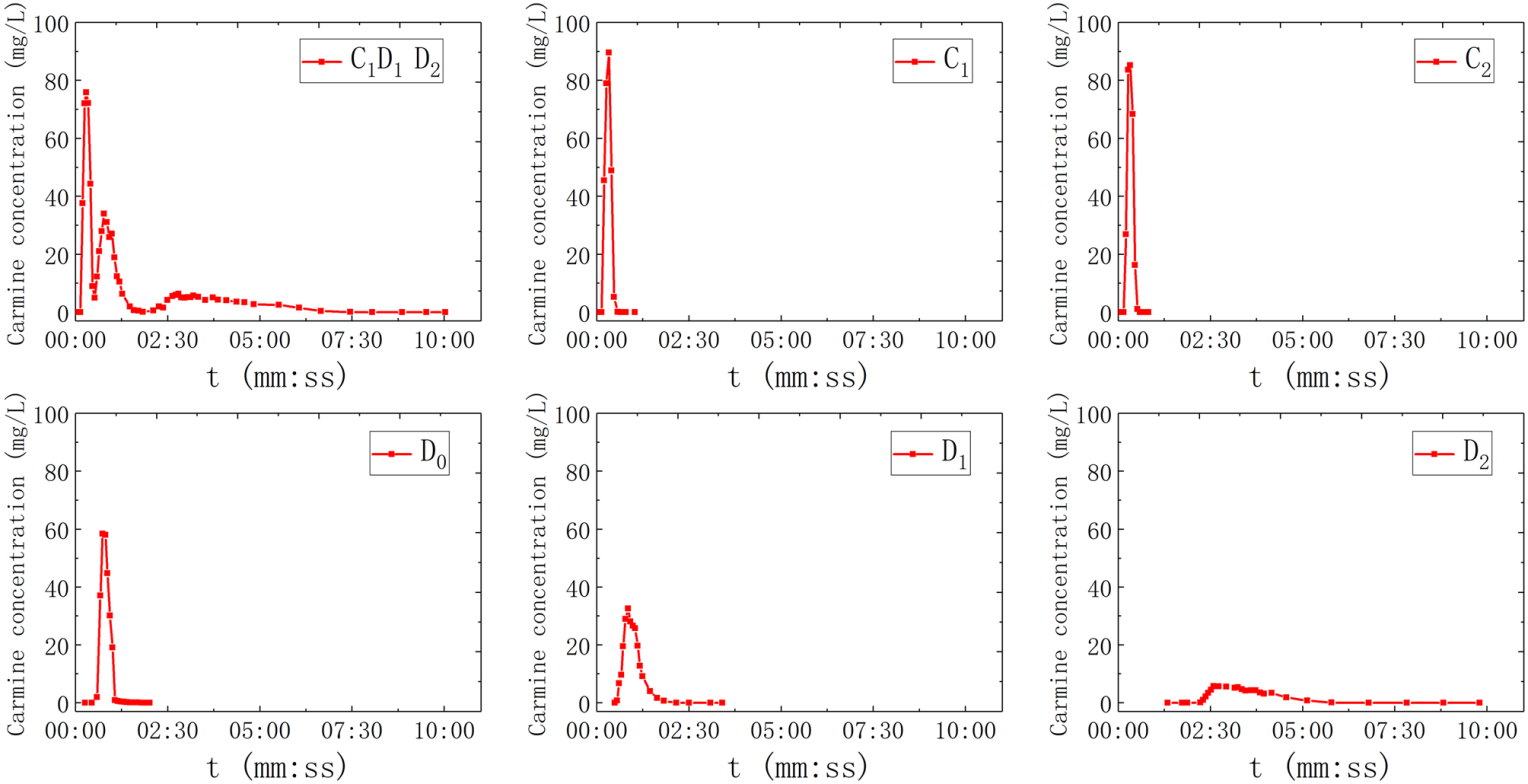
The BTC of solute transport under water-filling of conduit condition.

In this scenario, the water valves controlling all pipelines (C_1_, C_2_, D_0_, D_1_, D_2_) will be opened, and the water valves controlling fissures (F_1_, F_2_, F_3_, F_4_) will be closed to make the water flow in conduit saturated flow state. After adjustment and stabilization, the rainfall intensity was 3.8861 mm/h, and the outlet flow was 52.63 ml/s. As shown in Fig. 3, C_1_, C_2_, D_0_, D_1_, and D_2_ were respectively selected for single-point injection (20 ml), and C_1_–D_1_–D_2_ for multi-point injection, carmine solution (20 ml) was put into each point of C_1_, D_1_ and D_2_ at the same time. A total of 6 sets of solute transport tests under the condition of conduit saturation were simulated, and the BTCs are shown in Fig. 6.

In the simulation experiment of saturated water flow in the conduit, the BTC of single-point injection is a single-peak type, and that of multi-point injection is a multi-peak type (Fig. 6). The curves of C_1_ and C_2_ are completely symmetrical, and the peak concentration is the highest. In addition, the concentration of tracer rises rapidly and decreases rapidly in the process of transport. The retention time of the tracer in C_1_ is less than 62 s, and that of C_2_ is less than 50 s. However, compared with conduit conditions, the retention time of tracer in the doline is longer. The peak values of the BTC of three-point injection (C_1_–D_1_–D_2_) are staggered with each other, showing a three-peak type.

Based on the simulation results of the above three groups of experiments, the characteristic values of BTCs were summarized in Table 1. The dispersion parameters of each experiment group were calculated by using the two-dimensional dispersion model of the tracer injection of the instantaneous point.

**Table 1:** Characteristic parameters of solute transport in fissures–conduits saturated flow.

Saturated state	Injection point	*X* (m)	*t*_0_ (s)	*t*_m_ (s)	*c*_m_ (mg/L)	Rate of recovery (%)	*V* (m/s)	*D*_L_ (m^2^/s)
F–C-D	P	3.98	66	96	4.4785	31.46	0.02855	0.02168
	C_1_	1.88	8	31	33.8344	87.89	0.05099	0.00835
	C_2_	2.15	9	29	45.1534	99.00	0.06984	0.00449
	D_0_	2.01	55	84	21.10429	77.79	0.02175	0.00209
	D_1_	1.81	34	57	19.3865	70.08	0.02584	0.00485
	D_2_	1.73	20	170	3.8957	39.10	0.00837	0.00142
	F_1–2–3–4_	2.28	42	71	2.7914	21.56	0.01416	0.01474
	C_2_-D_2_	–	7	14	46.1963	74.42	–	–
				66	12.4540			
	F_1_-C_2_-D_2_	–	8	16	58.8650	60.72	–	–
				169	6.5337			
F	F_1_	2.28	35	46	18.6503	36.44	0.03523	0.01398
	F_2_	2.28	36	70	12.2699	67.38	0.02515	0.00750
	F_3_	2.28	45	71	8.7730	60.48	0.02472	0.00745
	F_4_	2.28	48	70	3.1902	27.44	0.02466	0.00792
	F_1–2_	2.28	39	66	14.7644	60.56	0.02606	0.00849
	F_1–2–3–4_	2.28	40	82	13.4663	63.51	0.02256	0.00541
C	C_1_	1.88	5	20	89.5399	94.65	0.08792	0.00553
	C_2_	2.15	10	19	85.2147	88.60	0.10850	0.00490
	D_0_	2.01	25	44	58.4049	91.35	0.04253	0.00306
	D_1_	1.81	27	51	32.5153	84.60	0.03248	0.00261
	D_2_	1.73	120	155	5.7975	55.75	0.00966	0.00121
	C_1_-D_1_-D_2_	–	5	18	75.8589	85.28	–	–
				47	34.0184			
				167	6.3497			

## Discussion

The purpose of the dispersion experiment is to study the temporal and spatial variation of pollutant concentrations in groundwater. The dispersion coefficient is an important hydrogeological parameter of solute transport, which represents the dispersion ability of porous media to a dissolved material at a certain velocity^27,28^. From the two-dimensional dispersion model, we can find that there are many parameters that affect the longitudinal dispersion coefficient. Therefore, the values of each parameter are correlated with the longitudinal dispersion coefficient to discuss solute transport process in karst aquifers.

### The analysis of factors influencing longitudinal dispersion coefficient

In this experiment, only one concentrated discharge point is set up, which is also the only discharge outlet and the sampling point of the tracer. The transport and diffusion of tracer mainly occur along the flow direction, so the longitudinal dispersion in the process of tracer transport is considered. The variation of longitudinal dispersion coefficient (*D*_L_) is analyzed from the linear distance (*x*) between the injection point and the receiving point, initial time (*t*_0_), peak time (*t*_m_), peak concentration (*c*_m_), average tracer transport velocity (*V*), and porosity (*p*) of aqueous media.

1. The correlation between the *x* and *D*_L_.

**Figure 7:**
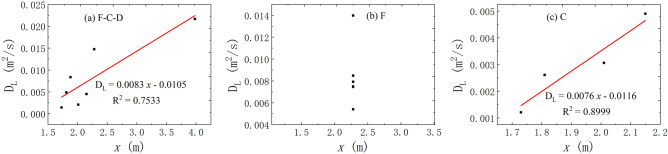
The correlation between the *x* and *D*_L_ in different flow condition.

According to the correlation analysis of the *x* and *D*_L_, Fig. 7 can find that the relationship between the two shows different correlation under the three flow states. There is a better linear positive correlation between the *x* and *D*_L_ under the condition of water-filling in fissure–conduit and conduit. However, the linear distance of each experiment is the same under the condition of fissure water-filling, and its dispersion coefficient is different due to the influence of other factors. Therefore, there are no correlation at this condition.

(2) The correlation between the *t*_0_ and *D*_L_.

**Figure 8:**
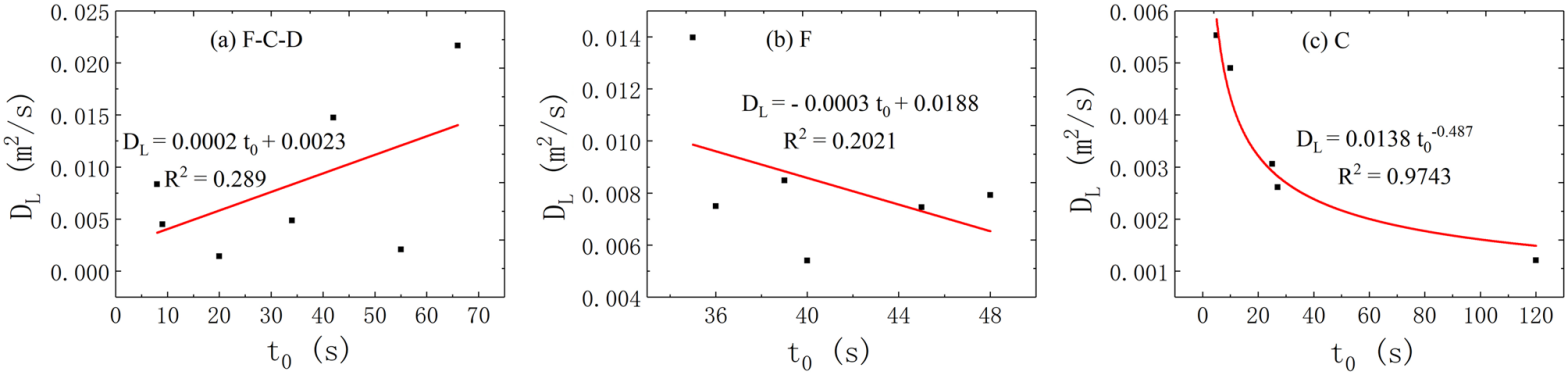
The correlation between the *t*_*0*_ and *D*_L_ in different flow condition.

About the correlation analysis of the *t*_*0*_ and *D*_L_, Fig. 8 illustrates different relationship between the two under the three flow states. The distribution of the *t*_*0*_ and *D*_L_ is scattered and there is insignificant correlation between the *t*_*0*_ and *D*_L_ in the water-filling test of fissure–conduit and fissure. However, there is a better power exponential function relationship between them under the condition of conduit water-filling.

(3) The correlation between the *t*_m_ and *D*_L_.

**Figure 9:**
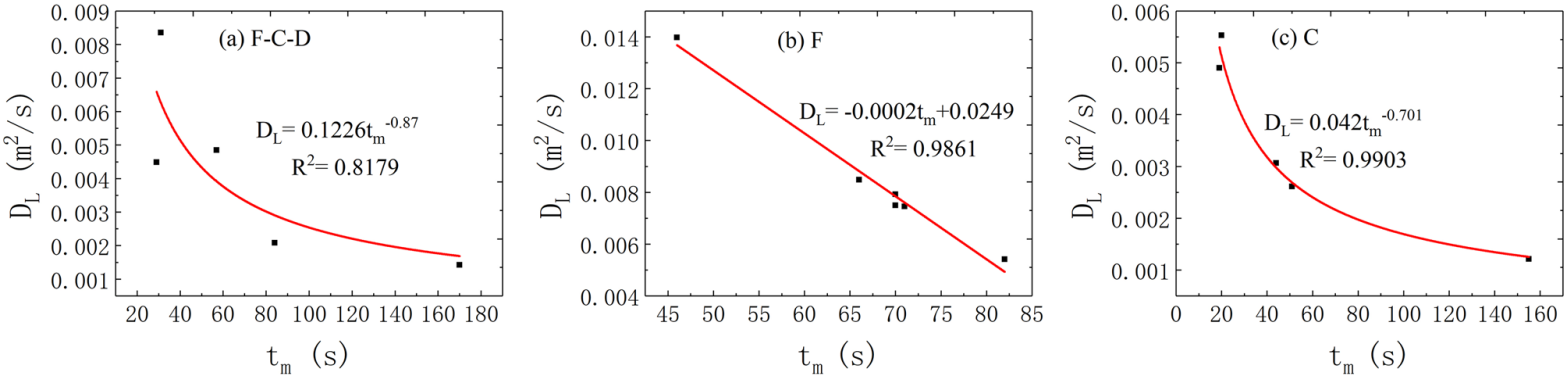
The correlation between the *t*_*m*_ and *D*_L_ in different flow condition.

Figure 9 demonstrates that the relationship between the two shows different correlation under the three flow states. The relationship between the *t*_m_ and *D*_L_ can be explained by power exponential function under the condition of fissure–conduit water-filling. Under the condition of conduit water-filling, it shows the same relationship. Otherwise, a linear relationship appears at the condition of fissure water-filling.

(4) The correlation between the *c*_m_ and *D*_L_.

**Figure 10:**
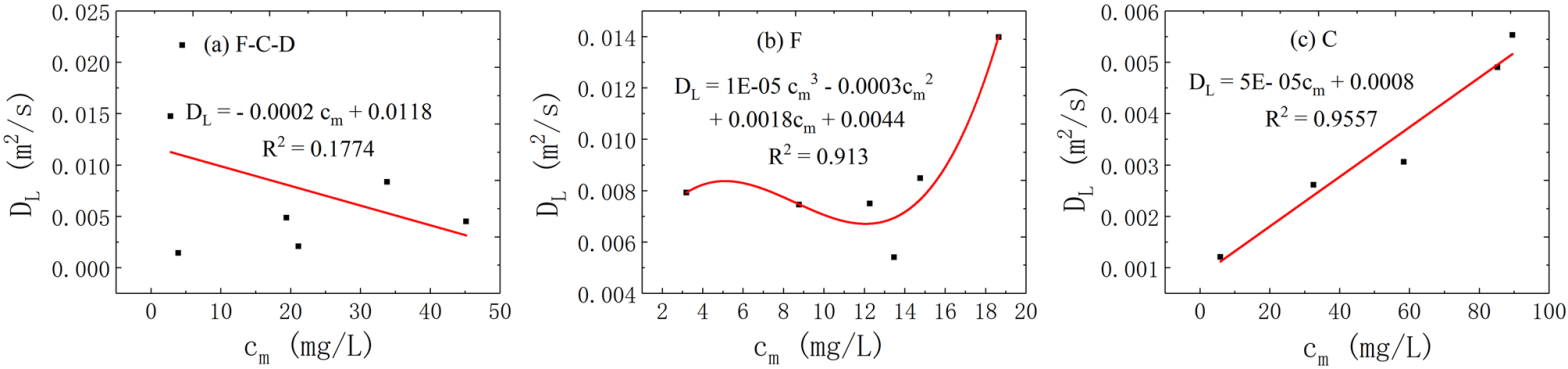
The correlation between the *c*_*m*_ and *D*_L_ in different flow condition.

The correlation analysis of the *c*_m_ and *D*_L_ is plotted in Fig. 10. It is observed that the relationship between the two shows different correlation under the three flow states. When the fissure–conduit is filled with water, the distribution of the relationship points between the *c*_m_ and *D*_L_ is scattered, and there is no obvious correlation. When the fissure is filled with water, the relationship between the two is a cubic polynomial. Then, the relationship between the two is linear when the conduit is filled with water.

(5) The correlation between the *V* and *D*_L_.

**Figure 11:**
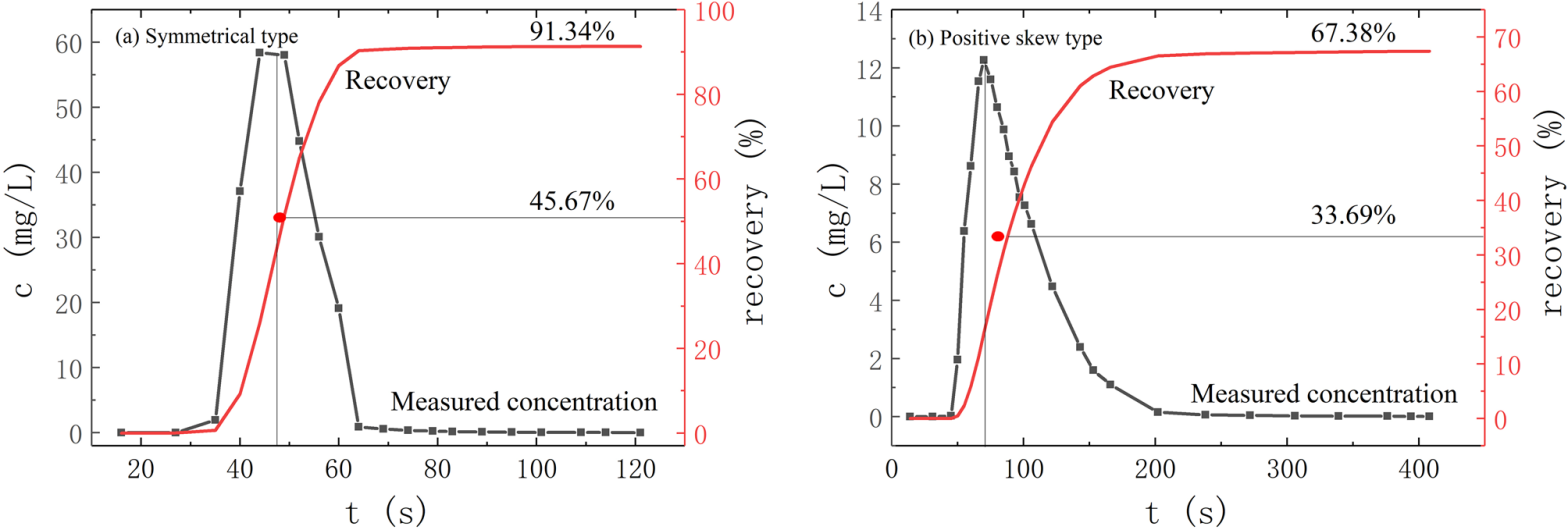
The method of BTC and cumulative recovery curve to determine concentration centroid.

In the tracer experiment, if the linear distance of solute transport is known, the average transport velocity of tracer can be estimated according to the average time of tracer transport. However, the average transport time of the tracer cannot be directly read from the breakthrough curve of solute transport. In general, the average transport time of tracer is between the peak time and the time of 50% tracer discharge^24^. Therefore, the average transport time can be estimated according to the centroid of the concentration of the BTC. In this experiment, the estimation of average transport time can be divided into two cases. In the first case, when the breakthrough curve is symmetrical, the average transport time can be adjusted appropriately, and the peak time can be taken; in the second case, when the breakthrough curve shape is positive skew type, the average transport time is adjusted to the half recovery time direction based on the peak time. By superimposing the breakthrough curve and the cumulative recovery curve on the same plot, the half recovery time of the tracer can be determined (Fig. 11).

According to the known linear transport distance and the average transport time estimated by the centroid method, the average transport velocity can be calculated (Table 2).

**Table 2:** The evaluation of mean velocity of tracer transport in fissure–conduit saturated flow.

Saturated state	Injection point	*X* (m)	t (s)	Concentration centroid velocity (m/s)	Two-dimensional dispersion velocity (m/s)	Relative error (%)	Average velocity (m/s)
F–C-D	P	3.98	136	0.02926	0.02855	2.44221	0.02891
	C_1_	1.88	34	0.05529	0.05099	7.78404	0.05314
	C_2_	2.15	29	0.07414	0.06984	5.79721	0.07199
	D_0_	2.01	84	0.02393	0.02175	9.10448	0.02284
	D_1_	1.81	57	0.03175	0.02584	18.62541	0.02880
	D_2_	1.73	170	0.01018	0.00837	17.75145	0.00927
	F_1–2–3–4_	2.28	136	0.01676	0.01416	15.53684	0.01546
F	F_1_	2.28	61	0.03738	0.03523	5.74430	0.03630
	F_2_	2.28	85	0.02682	0.02515	6.23904	0.02599
	F_3_	2.28	86	0.02651	0.02472	6.75789	0.02562
	F_4_	2.28	85	0.02682	0.02466	8.06579	0.02574
	F_1–2_	2.28	81	0.02815	0.02606	7.41842	0.02710
	F_1–2–3–4_	2.28	92	0.02478	0.02256	8.96842	0.02367
C	C_1_	1.88	20	0.09400	0.08792	6.46809	0.09096
	C_2_	2.15	19	0.11316	0.1085	4.11628	0.11083
	D_0_	2.01	44	0.04568	0.04253	6.89950	0.04411
	D_1_	1.81	51	0.03549	0.03248	8.48177	0.03399
	D_2_	1.73	155	0.01116	0.00966	13.45087	0.01041

**Figure 12:**
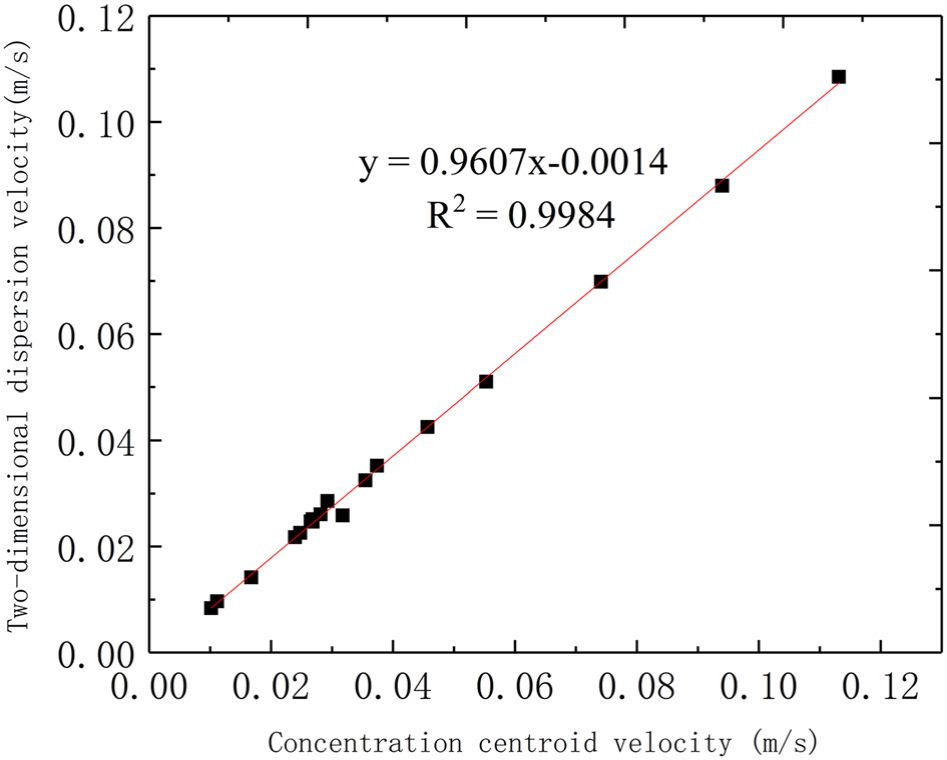
Correlation between concentration centroid method and two-dimensional dispersion numerical model for velocity estimation.

The average transport velocity of tracer estimated by concentration centroid method of BTC is close to the estimated value of two-dimensional dispersion numerical model for instantaneous point injection of tracer, and the estimated values of both fall on the straight line with an angle of 45° (Table 2, Fig. 12). Importantly, the results of the concentration centroid method of BTC verify the reliability of the application of the two-dimensional dispersion numerical model in this experiment.

**Figure 13:**
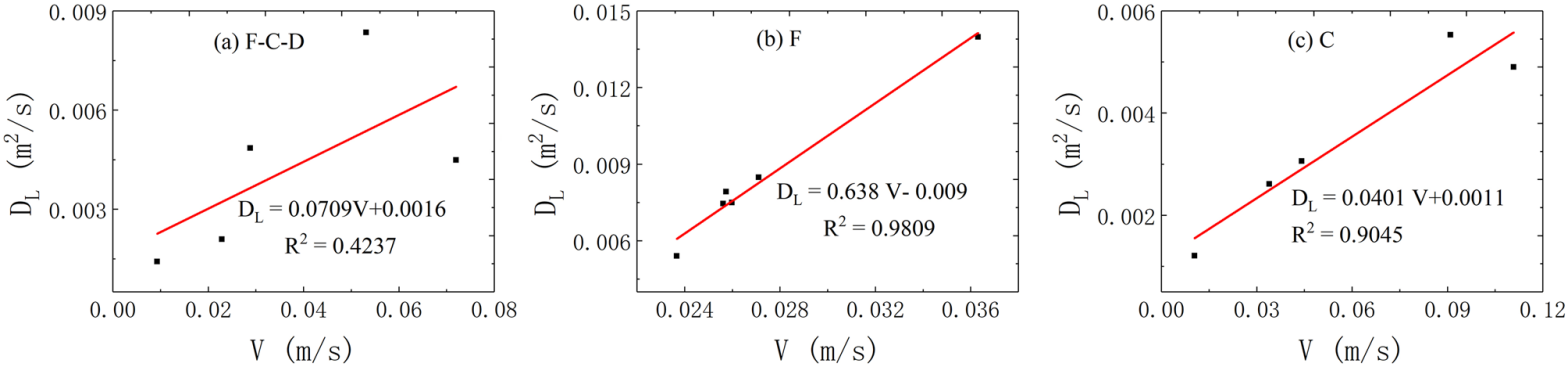
The correlation between the *V* and *D*_L_ in different flow condition.

Finally, Fig. 13 depicts the relationship between the *V* and *D*_L_ under the three flow states. It is found that the average transport time of tracer has a weak linear positive correlation with the *D*_L_ under the condition of fissure–conduit water-filling, while there is a significant linear positive correlation under the condition of fissure and conduit water-filling.

(6) The correlation between the *p* and *D*_L_.

The porosity in karst aquifer medium refers to the proportion of void volume to rock volume. There are two types of karst voids: primary void (micro void) and secondary void (macro void)^29^. The former is the result of diagenesis, while the latter is the result of tectonism, external force and karstification. Theoretically speaking, the result of karst process is the total transformation of voids from primary to secondary.

**Figure 14:**
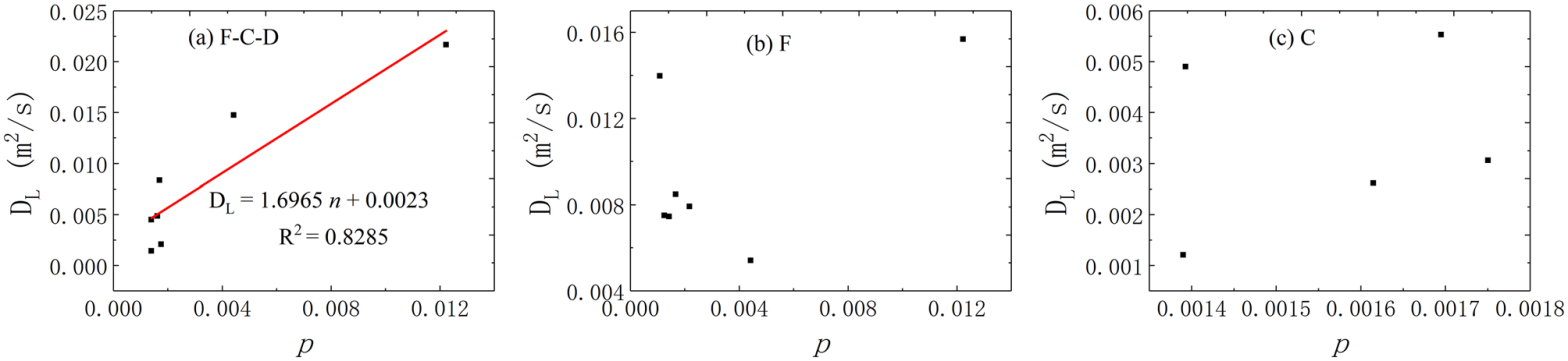
The correlation between the *V* and *D*_L_ in different flow condition.

The voids mainly involved in this experiment are macro. The effective void fraction of each aquifer channel is calculated by its void volume to the whole model volume.

17 $${\mathrm{p}}_{ei}= \frac{{V}_{ei}}{V}$$

where P_ei_ is the effective void fraction of the aquifer structure; *V*_ei_ is the effective volume of the aquifer structure; *V* is the space volume occupied by fissure–conduit the correlation analysis of the *p* and *D*_L_ is shown in Fig. 14. The results demonstrate that the porosity has a better linear correlation with the *D*_L_ under the condition of fissure–conduit water-filling. Meanwhile, it is also can be observed that the distribution of the relationship points is scattered and there is no obvious correlation between them under the condition of fissure and conduit water-filling.

In conclusion, the average transport velocity of the tracer has the most significant effect on the *D*_L_. In the saturated flow of fissure–conduit, the *D*_L_ has an approximately linear relationship with the *x* and *p*, a weak linear relationship with the *V*, and an approximate power exponential function relationship with the *t*_m_. Otherwise, no obvious relationship with the *t*_0_ and *c*_m_. In the saturated flow of fissure, the *D*_L_ has an important linear relationship with the *t*_m_ and *V*, and a vital cubic polynomial relationship with the *c*_m_, but no significant relationship with the *x*, *t*_0_, and *p*. In the saturated flow of conduit, a linear relationship exists between the *D*_L_ and the *x*, *c*_m_, *V*, *t*_0_, *t*_m_. Nevertheless, there is no obvious relationship with the *p*. In addition, the distribution of *D*_L_ is also different in different aquifer structures. The *D*_L_ varies from 0.00142 to 0.02168 m^2^/s when the fissure–conduit is saturated, 0.00541–0.01398 m^2^/s when the fissure is saturated, and 0.00121–0.00553m^2^/s when the conduit is saturated.

### The Gaussian multi-peak fitting

As mentioned above, there is no similar analysis of the relationship between the multi-peak phenomenon and various influencing factors. Due to the difference of dispersion effect in different aquifer structures, the BTCs of fissure–conduit and conduit filled with water present a multi-peak phenomenon, which is caused by a multi-path of groundwater flow system^30^. However, this phenomenon cannot be explained by the two-dimensional dispersion model of the tracer injection of the instantaneous point. These are C_2_–D_2_ (double peak), F_1_–C_2_–D_2_ (double peak) in Fig. 4, and C1–D1–D2 (triple peak) in Fig. 6. The Gaussian multi-peak fitting has achieved reliable results in other disciplines, but it is seldom used in the analysis of solute transport in a karst aquifer^31,32^. Therefore, in order to reflect the overall shape and change trend of the BTC of multi-peak, we carried out the Gaussian multi-peak fitting to study it. Gauss multi-peak fitting is a more accurate way to reflect the overall shape and the trend of BTC. It is formed by the linear combination of multiple Gauss functions, and the fitting accuracy can be increased or reduced by adjusting the number of Gauss functions.

The fitting function is a linear combination of several Gaussian functions:

18 $$G\left(x,a,b,c\right)=\sum_{i=1}^{n}{a}_{i}exp\left(-{\left(\frac{x-{b}_{i}}{{c}_{i}}\right)}^{2}\right)$$

where *a*_i_, *b*_i_, and *c*_i_ are the parameters to be solved. According to the measured data, the corresponding parameters to be solved can be obtained by using the least square method (Eq. 19), that is, the *a*_i_, *b*_i_ and *c*_i_ that minimize the mean square error *Q* (*a*, *b*, *c*), and then the Gaussian multimodal fitting function can be determined.

19 $$MINQ\left(a,b,c\right)=\sum\limits_{j=1}^{m}{\left(\sum\limits_{i=1}^{n}{a}_{i}exp\left(-{\left(\frac{{x}_{j}-{b}_{i}}{{c}_{i}}\right)}^{2}\right)-{y}_{j}\right)}^{2}$$

**Figure 15:**
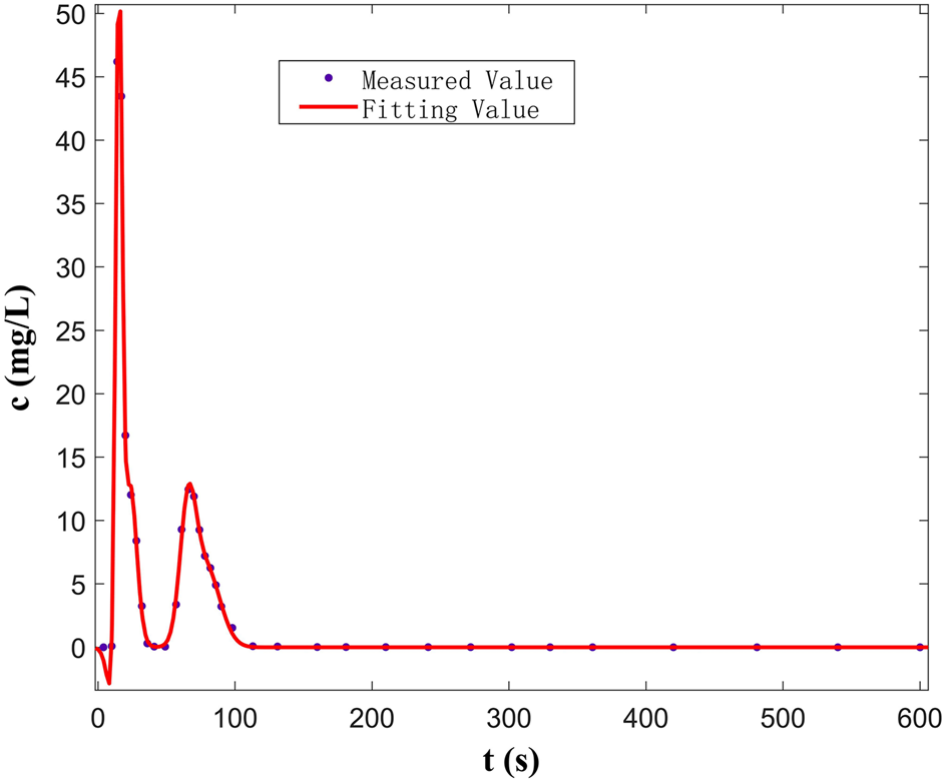
Curve fitted by Gauss multi-peaks method (C_2_-D_2_).

According to the Gauss multi-peak fitting results of C_2_–D_2_ (double peak) when the fissure–conduit is saturated, the best fitting curve is determined by the linear combination of seven Gaussian functions (Fig. 15). The correlation coefficient is 0.9999, the sum of error squares is 0.304, and the RMSE is 0.1473. The optimal solutions of various parameters are shown in Table 3.

**Table 3:** Parameters determined by Gauss multi-peaks method with 95% confidence bounds (C_2_–D_2_).

Parameters of Gauss linear function	The least square optimal solution of each parameter		
(a1,b1,c1)	73.86	14.36	3.814
(a2,b2,c2)	− 33.98	12.55	3.519
(a3,b3,c3)	− 12.58	67.16	4.736
(a4,b4,c4)	11.92	23.87	7.02
(a5,b5,c5)	− 12.62	72.34	5.637
(a6,b6,c6)	28.44	68.65	7.67
(a7,b7,c7)	5.551	79.59	15.56

**Figure 16:**
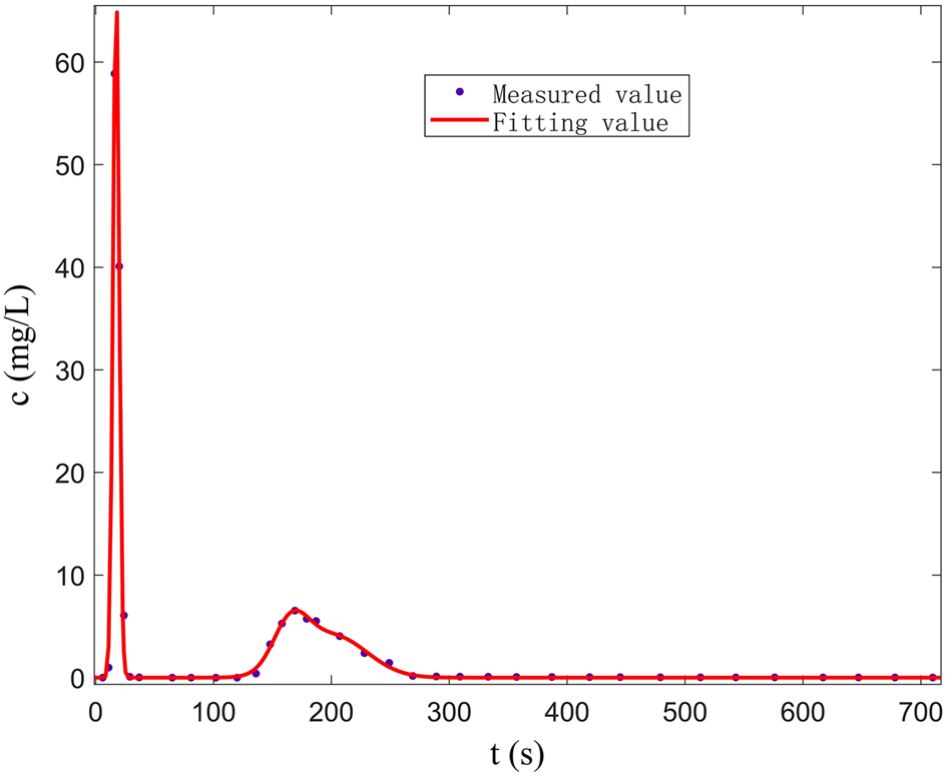
Curve fitted by Gauss multi-peaks method (F_1_-C_2_-D_2_).

In Fig. 16, the best-fitting curve of F_1_–C_2_–D_2_ (double peak) is plotted by the linear combination of three Gaussian functions in the fissure–conduit saturated flow. The correlation coefficient is 0.9961, the sum of square error is 18.32, and the RMSE is 0.8236. We can observe that the measured values are consistent with the fitting curve. The optimal solutions of various parameters are shown in Table 4.

**Table 4:** Parameters determined by Gauss multi-peaks method with 95% confidence bounds (F_1_–C_2_–D_2_).

Parameters of Gauss linear function	The least square optimal solution of each parameter		
(a1,b1,c1)	67.83	17.4	3.638
(a2,b2,c2)	4.486	165.5	20.91
(a3,b3,c3)	4.084	201.2	40.83

**Figure 17:**
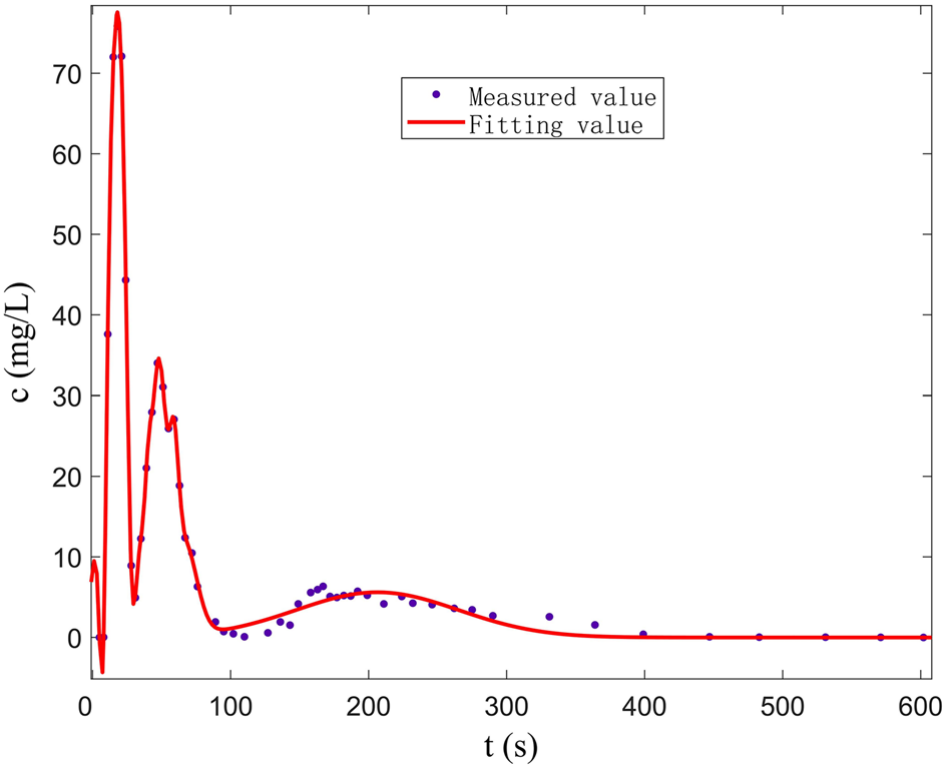
Curve fitted by Gauss multi-peaks method (C_1_-C_2_-D_2_).

Also, based on the Gauss multi-peak fitting results of C_1_–C_2_–D_2_ (triple peaks) in the conduit saturated flow, it is manifest that the measured values are consistent with the fitting curve. The best fitting curve is determined by the linear combination of eight Gaussian functions (Fig. 17). The correlation coefficient is 0.9979, the sum of square error is 37.22, and the RMSE is 1.153. The optimal solution of each parameter is shown in Table 5.

**Table 5:** Parameters determined by Gauss multi-peaks method with 95% confidence bounds (C_1_–C_2_–D_2_).

Parameters of Gauss linear function	The least square optimal solution of each parameter		
(a1,b1,c1)	78.87	18.87	12.78
(a2,b2,c2)	32.5	47.98	7.123
(a3,b3,c3)	− 38.87	7.542	3.71
(a4,b4,c4)	15.83	58.86	4.784
(a5,b5,c5)	− 38.94	27.9	5.133
(a6,b6,c6)	9.406	40.4	3.446
(a7,b7,c7)	11.45	66.4	12.61
(a8,b8,c8)	5.614	206.3	83.37

The fitting results of the multi-peak BTCs in the above three groups of experiments are consistent with the measured values, and the fitting effect is better. The peak concentration of the first peak is large and the peak appearance time is short (Fig. 15). The curve shape is sharp and thin, which is mainly controlled by the C_2_. During the experiment, it can be observed that the carmine solution first migrates to the outlet in the C_2_. However, the peak concentration of the second peak is small, and the peak appearance time lags, and the curve shape is short, which is mainly controlled by the D_2_. Furthermore, the peak concentration of the first peak is mainly controlled by the C_2_ (Fig. 16). The peak concentration of the second peak is mainly controlled by the F_1_ and D_2_, and the solute transport process in F_1_ and D_2_ overlaps. Therefore, a BTC of the peak is formed. Also, there are three peaks in the tracer test of three-point injection. The main peak is controlled by the C_1_, the secondary peak is mainly controlled by the C_2_; the minimum peak is mainly controlled by the D_2_. Compared with other single combination aquifer structure, only the BTC of single-peak appears. Therefore, we can find that the BTC of multi-peak is related to the type diversity of aquifer media. Different aquifer media have different velocity and time of solute transport, which is the mechanism of forming BTC of multi-peak.

## Conclusion

In this experiment, we simulated the solute transport experiments affected by different injection methods in the saturated flow of karst aquifer. The main factors affecting the solute transport are the aquifer structure and water flow state of the aquifer. Under different conditions, the velocity of solute transport is also affected, so the breakthrough curves obtained are different in the dynamic responses of the linear distance (*x*) between the injection point and the receiving point, initial time (*t*_0_), peak time (*t*_m_), peak concentration (*c*_m_), average tracer transport velocity (*V*), and porosity (*p*) of aqueous media. the following conclusions are deduced based on the present study:

1. Under different water filling conditions, the influence of various factors on the longitudinal dispersion coefficient (*D*_L_) is different. ①Under the condition of fissure water-filling, the *D*_L_ has a weak linear correlation with the *t*_0_, a positive linear correlation with the *t*_m_ and the *V*, a cubic relationship with the *c*_m_) and a poor correlation with the *p.* ②When the pipeline is filled with water, the *D*_L_ has a positive linear correlation with the *x* between the injection point and the receiving point, a negative power function correlation with the *t*_0_ and *t*_m_, a linear correlation with the *V* and *c*_m_, and a poor correlation with the *p.* Under the condition of water filling in fissure–conduit, the *D*_L_ has a positive linear correlation with the *x* and *p* between the injection point and the receiving point, but a poor correlation with the *t*_0_ and *c*_m_, a negative power function with the *t*_m_, and a weak linear correlation with the *V*. In the process of analyzing the correlation between the *V* and the *D*_L_, the centroid method is used to calculate the average transport velocity of tracer, which verifies the reliability of the application of the two-dimensional numerical model in this experiment.
2. The appearance of the multi-peak phenomenon is related to the type of aquifer medium, and BTC of single type aquifer medium is single-peak. Besides, the difference of velocity in different aquifer media, which leads to the appearance of a multi-peak phenomenon. Therefore, the type of BTC can be confirmed by the tracer experiment. Generally, in the multi-point injection solute transport experiment, according to the measured data, Gaussian multi-peak fitting can be used to reflect the overall shape and change trend of the multi-peak BTC.
3. This paper has a very important reference significance for the simulation of solute transport and the solution of pollutant transport in karst aquifer medium. Although some breakthroughs have been made in the study of fissure–conduit hydrodynamic dispersion process, there are still some deficiencies in the complex karst aquifer system. At the same time, the injection mode of tracer in this experiment is the instantaneous injection, while the pollutants in the objective environment are only non-instantaneous emissions. Therefore, the following work should further simulate the solute transport of complex transformation injection mode (continuous injection, equal concentration intermittent injection, and unequal concentration intermittent injection) and study its multiple pollution process.
